# Supplementary material for: Different Oncologic Outcomes According to Margin Status (High-Grade Dysplasia vs. Carcinoma) in Patients Who Underwent Hilar Resection for Mid-Bile Duct Cancer
Source: Cancers (Basel). 2023 Oct 26;15(21):5166. doi: 10.3390/cancers15215166 (PMC10650487; doi:10.3390/cancers15215166)
Supplement: Supplementary file 1 [file cancers-15-05166-s001.zip › cancers-2567371-supplementary.pdf]

cancers

MDPI

Supplementary Table S1. location of positive margin for R1 patients (n=25).

Location	R1 HGD (n=9)	R1 Carcinoma (n=14)
PROXIMAL MARGIN	5 (55.6)	8 (57.1)
DISTAL MARGIN	4 (44.4)	6 (42.9)
BOTH	0	0

HGD, High grade dysplasia.

Supplementary Table S2. Uni-and multivariate analysis identifying factors affecting disease free survival (n=149).

VARIABLE	PATIENTS (N)	5Y DFS (%)	UNIVARIATE ANALYSIS			MULTIVARIATE ANALYSIS		
			HR	95% CI	*P*	HR	95% CI	*P*
SEX								
MALE / FEMALE	104 / 45	27.9 / 40.5	0.860	0.562-1.315	0.486			
AGE								
≤ 65/> 65	56 / 93	44.9 / 23.9	1.540	1.010-2.348	0.045	1.302	0.843-2.011	0.234
BMI								
≤ 25/> 25	106 / 43	26.2 / 46.3	0.691	0.440-1.086	0.109			
ASA SCORE					0.239			
I	18	9.4						
II	113	37.1	0.699	0.399-1.227	0.212			
III/IV	18	18.9	1.038	0.501-2.154	0.919			
PREOP CA19-9								
≤ 35/> 35	84 / 65	39.2 / 21.7	2.580	1.748-3.809	<0.001	2.026	1.327-3.094	0.001
T-STAGE					<0.001			0.078
T1	47	56.8						
T2	91	21.2	2.617	1.621-4.226	<0.001	1.778	1.059-2.983	0.029
T3/4	11	18.9	2.535	1.124-5.719	0.025	1.948	0.824-4.606	0.129
N STAGE								
N (-) / N (+)	107 / 42	38.2 / 15.2	2.084	1.390-3.123	<0.001	1.573	1.029-2.405	0.036
MARGIN					0.344			
R0	126	35.2						
R1 HGD	9	14.8	1.482	0.643-3.415	0.356			
R1 CARCINOMA	14	9.8	1.458	0.794-2.675	0.224			
COMPLICATIONS								
NO / YES	133 / 16	34.2 / 13.5	1.503	0.832-2.715	0.177			
CHEMOTHERAPY								
NO / YES	135 / 14	32.8 / 16.3	1.458	0.756-2.810	0.260			

DFS, Disease free survival; HGD, High grade dysplasia.
